# Women in Surgery: An Analysis of Mental Health, Stress Perception and Resilience

**DOI:** 10.1007/s43465-025-01448-9

**Published:** 2025-06-19

**Authors:** Suzen Agharia, Hannah de Wet, Claudia Di Bella

**Affiliations:** 1 Faculty of Medicine, Nursing and Health Sciences, Monash University, Melbourne, VIC Australia; 2 School of Nursing and Midwifery, Faculty of Health, Deakin University, Melbourne, VIC Australia; 3 Orthopaedic Department, Sarcoma Unit, St Vincent’s Hospital Melbourne, Melbourne, VIC Australia; 4 Department of Surgery, The University of Melbourne, Melbourne, VIC Australia

**Keywords:** Women in surgery, Mental health, Stress, Resilience, Coping

## Abstract

**Purpose:**

Although the representation of women in surgical fields is increasing, challenges such as gender bias, demanding work conditions, and balancing career with personal responsibilities persist. This study aims to investigate the stressors affecting female surgeons, assess their impact, and explore the resilience strategies employed to mitigate these effects.

**Methodology:**

A literature review was conducted using PubMed to identify peer-reviewed articles on mental health, stress perception, and coping among female surgeons. From the initial search, 49 articles were identified, with additional studies manually added from the references of reviewed literature.

**Results:**

The review highlighted key stressors for female surgeons, including workplace culture, gender bias, and sexual harassment. Resilience-building strategies, such as mindfulness and institutional support, were found to positively impact mental health and stress management. Female surgeons face unique stressors stemming from the demands of the profession, systemic gender biases, and entrenched cultural norms, leading to significant mental health challenges such as burnout and anxiety. While personal coping mechanisms, like mindfulness, show some efficacy in mitigating these issues, institutional-level challenges remain largely unaddressed. Gendered expectations and exclusionary workplace cultures continue to hinder progress, underscoring the need for structural reforms to improve the professional environment and promote the well-being of female surgeons.

**Conclusion:**

While resilience strategies offer some relief, substantial policy reform and a shift in workplace culture are crucial to creating a more supportive and equitable environment for women in surgery.

## Introduction

Over recent decades, there has been an increase in the number of women entering surgical specialties. This reflects broader societal changes towards gender equality while dismantling the traditional barriers in male-dominated professions. Despite this progress, female surgeons encounter unique challenges related to the demanding nature of the role and systemic issues, including discrimination, gender bias, gender inequality, and the difficulty of balancing professional and personal responsibilities. These challenges are both real and perceived. This review examines the specific stressors affecting women in surgery, their impact on mental health, and the coping mechanisms and resilience strategies they employ.

## Methods

### Literature Review Process

A comprehensive literature review was performed to explore the mental health, stress perception, and resilience of women in surgery. We systematically searched the PubMed database to identify peer-reviewed articles, clinical studies, and systematic reviews that addressed the specific challenges faced by female surgeons and their impact on mental health, stress, and resilience.

### Search Strategy

The search terms focused on key themes, including “women in surgery,” “mental health,” “stress perception,” and “resilience.” This is outlined in Table 1 in further detail.

**Table 1:** Outlines the search terms used for literature review on PubMed

Theme	Search terms	Search yield
Mental health and women in surgery	(“women in surgery”[Title/Abstract] OR “female surgeons”[Title/Abstract] OR “women surgeons”[Title/Abstract]) AND (“mental health”[Title/Abstract] OR “psychological wellbeing”[Title/Abstract] OR “Burnout”[Title/Abstract])	47 results
Stress perception in female surgeons	(“women in surgery”[Title/Abstract] OR “female surgeons”[Title/Abstract] OR “women surgeons”[Title/Abstract]) AND (“stress perception”[Title/Abstract] OR “workplace stress”[Title/Abstract] OR “job stress”[Title/Abstract] OR “work related stress”[Title/Abstract] OR “occupational stress”[Title/Abstract])	2 results
Resilience and coping in female surgeons	(“women in surgery”[Title/Abstract] OR “female surgeons”[Title/Abstract] OR “women surgeons”[Title/Abstract]) AND (“Resilience”[Title/Abstract] OR “Coping”[Title/Abstract] OR “Adaptation”[Title/Abstract] OR “emotional resilience”[Title/Abstract])	15 results

The studies were included if they were published in peer-reviewed journals, discussing mental health, stress perception, and resilience of female surgeons and outlined specific challenges faced by women in surgery, including, but not limited to, gender bias, workplace culture, stress, burnout, and resilience strategies. Studies not published in English and if they did not explore the relevant themes were excluded. Non-peer-reviewed articles, conference abstracts, and opinion pieces were also excluded. The search was not limited by a time frame to identify trends in the forms of stressors among female surgeons and coping mechanisms across time.

### Data Collection and Synthesis

Our search strategy yielded 64 results, which were screened by titles and abstracts for relevance by two assessors. Full-text reviews were then performed for articles meeting inclusion criteria, and further 10 articles were included based on references from the reviewed literature. The full texts of each article were reviewed to extract data on the specific stressors faced by female surgeons, mental health outcomes associated with these stressors, coping strategies and resilience-building techniques, and the institutional and personal interventions recommended to support women in surgery. A quality assessment of the included articles was not performed.

**Fig. 1:**
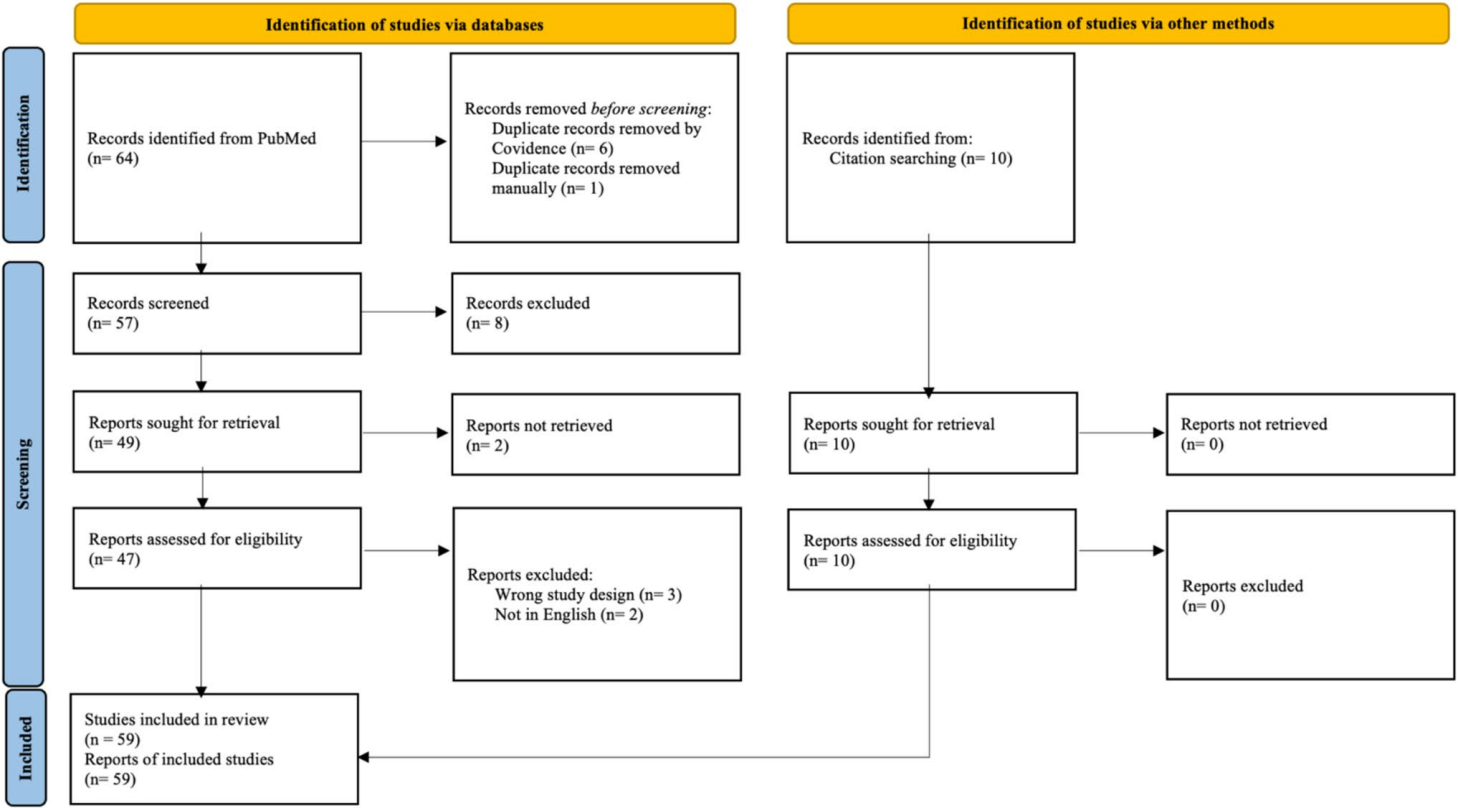
PRISMA flowchart illustrating the study selection process for the literature review, detailing the identification, screening, eligibility assessment, and inclusion of studies from PubMed and other sources

Once the relevant studies were identified and assessed, the data were synthesised into a comprehensive narrative, focusing on the common themes and differences between the studies. This review synthesises the findings in a narrative format organised by thematic categories, including stressors, coping mechanisms, and resilience-building strategies. Although we did not perform a formal meta-analysis, we adhered to PRISMA guidelines in the selection of studies and reporting of results to ensure a comprehensive and unbiased summary of the literature (Fig. 1).

## Results

The literature review identified 59 studies that fulfilled the inclusion criteria, focusing on mental health, stress perception, and resilience among female surgeons. The findings were categorised into eight thematic areas: stressors, burnout and emotional exhaustion, workplace culture and gender bias, barriers in training and academia, harassment, mental health challenges, maladaptive coping strategies, and resilience and coping. Among the included studies, 20 investigated stressors impacting female surgeons, including burnout and emotional exhaustion. Six studies explored workplace culture and gender bias, while five addressed barriers in training and academia. Thirteen studies examined harassment, six focused on mental health challenges, and six analysed maladaptive coping strategies. In addition, 13 studies investigated resilience and coping mechanisms. However, it should be noted that several studies were relevant to and discussed across multiple thematic areas.

## Discussion

### Stressors

Excessive stress among surgeons impairs patient outcomes and increases the risk of burnout and self-harm, including substance abuse and suicide [1]. Burnout in surgical specialties is notably more prevalent among women, primarily due to increased work-related stress and personal demands. A 2011 cross-sectional study identified gender as a significant risk factor for burnout, revealing that women were more likely to experience burnout and depression compared to their male colleagues, even with male and female colleagues working the same hours [2]. These findings highlight the need for interventions such as mentorship programs and flexible scheduling, and financial literacy to reduce systemic pressures [3, 4]

Similarly, female surgeons, and particularly female trainees, report these symptoms alongside reduced personal accomplishment, underscoring the need for institutional support and resilience strategies to mitigate these effects [5, 6]. A survey of 110 hand surgeons found that women reported significantly higher stress than men (55.1 vs. 44.3%), even during their time away from work on holidays. It is also reported that they experienced greater negative impacts on their personal relationships [7]. These findings align with research suggesting that burnout results from inadequate stress management within the workplace. It is argued that piecemeal solutions, like resilience training, may only help those already nearing burnout [6]. 

Female orthopaedic surgeons report higher rates of depression and anxiety, with the lack of supportive infrastructure in the surgical field being a major factor. Interventions, such as mental health resources and peer support groups, can help surgeons manage stress and maintain their well-being [8]. Gender disparities in work–home conflicts, burnout, and career satisfaction identified differences between male and female surgeons. Female surgeons, often younger with fewer children, face more work–home conflicts, prioritising work over personal life despite similar hours to their male counterparts. This leads to higher burnout, depression, and lower work–life satisfaction [9–11]. Female surgeons experience higher rates of pregnancy complications and infertility than the general population, with infertility rates at 32% compared to 10.9%. With more women entering into surgical training, the stress of balancing career and family becomes a significant issue, often forcing women to choose between their career and starting a family or take higher risks to continue working [9, 10]. While both genders face similar burnout risks, female surgeons tend to sacrifice personal lives for their careers, increasing stress and psychological strain [11].

### Burnout and Emotional Exhaustion

Burnout among female orthopaedic surgeons demonstrates persistent systemic issues established in the field. Studies investigating the relationship between career burnout and gender equity barriers have shown notable links between burnout and gender bias. A survey of 218 female Canadian orthopaedic surgeons showed that 50% experienced burnout, with major contributing factors including male privilege, devaluation, and disproportionate constraints. Meanwhile, 77.1% reported overall job satisfaction. Burnout was negatively correlated with age and job satisfaction, indicating that younger surgeons face more challenges [12]. Female surgeons also dedicated more time to documenting patient encounters and writing longer notes than their male counterparts, potentially contributing to burnout and pay disparities [13]. Burnout levels continue to be elevated, making it crucial to address burnout and foster a healthier, more sustainable career path for female surgeons [12, 14, 15]. This is further compounded among women balancing time between the public and private sectors [16]. Introducing more thorough equality and diversity training across all levels of surgery could help address both conscious and unconscious biases in the field. By focusing more on gender equality and raising awareness of biases, we can foster a more inclusive environment where all surgeons, regardless of gender, feel equally supported [17].

Emotional tolls are also starkly gendered. A study on compassion, fatigue and burnout among surgeons revealed that female surgeons exhibited higher burnout scores and greater compassion fatigue compared to males [18]. Among trauma surgeons, female practitioners reported lower compassion satisfaction and increased burnout than peers in other specialties. These findings highlight an intensified emotional burden, particularly in high-stakes fields like trauma surgery, underscoring the need for targeted support.

A survey of 335 surgeons across institutions explored gender disparities in stress distribution among surgeons [19]. Their findings revealed that women exhibited significantly higher maximum stress scores, regardless of parental status. Although more female surgeons had children under 18, this factor did not account for the gap in stress, challenging the common assumptions that family responsibilities are the primary driver of stress and burnout. Rather, workplace culture emerged as a major contributor, particularly in male-dominated fields such as orthopaedics, where exclusionary norms persist [19, 20].

### Workplace Culture and Gender Bias

Beyond individual stress, systemic factors like workplace culture amplify these challenges. In surgery, cultural norms prioritising toughness, endurance, and assertiveness pressure female surgeons to conform for acceptance, particularly in traditionally male-dominated specialties. This tension between personal identity and professional expectations is further compounded by gender-based biases. For instance, recent studies show that female surgeons shared experiences of being perceived as less competent or inferior to their male counterparts, who were seen as more equipped for the demanding nature of surgery [21, 22].

Stereotypes about surgical competence further complicate this for female surgeons. The notion that women lack the grit, perseverance, and passion for long-term goals needed for surgery is persistent despite evidence showing no gender difference in grit [23]. However, this stereotype endures, suggesting that external biases continue to undermine women’s perceived competence in the field. Patient assumptions that physical strength is essential in certain specialties, such as orthopaedics, further reinforce misconceptions that favour men, thereby creating barriers to diversity in the field [24]. These biases undermine confidence and lead to professional isolation, with studies showing that female surgeons often feel dismissed by their male colleagues [25]. As such stereotypes persist, gaining acceptance and recognition within the field remains a continuous challenge for female surgeons, impeding their career progression. In addition, research among female surgical residents has shown that those who strongly internalised these stereotypes experienced poorer psychological health compared to their peers who were less affected [26].

### Barriers in Training and Academia

Gender inequities in training persist, particularly in leadership and academic opportunities. A study in the United States found that women hold only 19% of full-time faculty positions in orthopaedic surgery and just 6.7% of endowed professorships at the top 100 academic centres, compared to 93.3% for men [27]. Many early-career female surgeons face challenges in developing their clinical practices. In clinical practice, women often receive fewer and less complex referrals than men, and they face additional barriers such as gender-based discrimination, salary gaps, and unequal resource allocation [28]. While this study was conducted in the United States, the literature indicates that trends of gender disparity in academic and leadership roles are observed globally, highlighting a broader challenge within the surgical field. Studies from various regions, such as Australia and Pakistan, also report similar patterns of underrepresentation and inequity, suggesting that these issues may be widespread, though potentially underreported in some areas due to systemic barriers or limited research focus [29, 30]. Gender concordance with patients and referring physicians can help women grow their practices. Institutions should recognise these barriers and make changes to support all surgeons, especially those early in their careers [28]. Similarly, at events like the Royal Australian College of Surgeons Annual Scientific Congress, women remain underrepresented and are mainly limited to sessions where conveners and chairs are women [29].

In academia, female surgeons continue to be underrepresented as speakers and authors, creating an environment that undervalues their academic contributions [29]. Similarly, there are reports that while female orthopaedic residents produce a comparable number of first-author publications as their male peers, they are less likely to secure primary authorship on high-impact research papers and tend to have fewer total publications [31]. These disparities create obstacles to advancements in personal careers and reinforce gender biases in academia. With the use of equitable mentorship and leadership, the gaps can be bridged and enable women to reach their full professional and academic potential.

### Harassment

Harassment is frequently perpetuated by poor workplace culture, representing a distinct form of misconduct. While there are cultural norms that inactively encourage inequalities, harassment is an active behaviour that can result in quite serious psychological and legal repercussions. Harassment remains a significant issue within the surgical profession, with implications for burnout, stress, and diminished professional satisfaction. A national survey of general surgery residents found that 19.9% of female respondents experienced sexual harassment, compared to only 0.9% of male respondents [32]. Attending surgeons were identified as the primary perpetrators in 27.2% of cases, reflecting the influence of power dynamics within surgical hierarchies [32–34]. Similar findings were observed in orthopaedic surgery, where 81% of women reported experiencing some form of discrimination, bullying, or sexual harassment, with many identifying harassment as a major stressor contributing to occupational dissatisfaction [33, 35–37]. In cardiothoracic surgery, 81% of women reported sexual harassment versus 46% of men, yet less than 5% of incidents reached governing boards due to bystander inaction [38].

Global data further highlights the prevalence and impact of harassment. In Pakistan, a study of female surgeons found that 57.5% of female surgeons faced harassment, 64% verbal and 45.9% mental, with 91.5% unreported due to systemic barriers. This study also identified a strong correlation between harassment and severe burnout, underscoring the need for institutional support [30, 37]. Microaggressions, subtle yet pervasive, compound these issues [34, 39]. An American College of Surgeons survey found that 67.4% of respondents experienced microaggressions, with women being 15.9 times more likely to report these incidents than men [35]. Unsurprisingly, these microaggressions contribute to feelings of exclusion, stress, and career regret among female surgeons [33, 34, 36, 40, 41].

The harassment exacts a profound psychological toll. Women reporting sexual harassment were more likely to experience burnout and suicidal ideation [32, 35, 39]. It has been noted that women who experience harassment report higher levels of occupational distress and are more likely to consider leaving their profession [42]. Fear of repercussions was identified as a barrier to reporting harassment, with participants noting concerns about potential negative career impacts and professional isolation [33, 36]. Women who experienced harassment reported reluctance to pursue formal complaints due to concerns about professional retaliation, stigmatisation, or being labelled as troublemakers. These fears were exacerbated by the lack of independent and supportive reporting pathways [33]. Furthermore, female trainees often tolerate sexualised banter to fit masculine norms, a pressure exacerbated by women’s underrepresentation in leadership, limiting advocacy for change [43].

Harassment in surgery represents a critical challenge with far-reaching implications for the mental health, career satisfaction, and retention of female surgeons. The persistence of such behaviour reflects deeper structural inequalities that require targeted reforms. Comprehensive interventions that combine robust policies, cultural change, and individual support mechanisms are necessary to mitigate the impact of harassment and promote the well-being of women in surgery.

### Mental Health Challenges

Mental health challenges among surgeons extend to severe conditions such as depression and suicidal ideation. A survey of 661 practising orthopaedic surgeons across subspecialties found that 23.6% had experienced active suicide ideation, with 5% formulating a specific self-harm plan [44]. The study identified several demographic and practice-related risk factors associated with depression and suicidal ideation, including younger age, divorce, and subspecialties such as adult reconstruction and foot and ankle surgery. Other factors such as gender, relationship status, and parental status were significant predictors of mental health outcomes. This emphasises the need to prioritise emotional well-being in orthopaedics and normalising discussions surrounding depression and suicidality to provide support for affected individuals [44].

Female orthopaedic surgeons experience higher rates of depression and anxiety due to the lack of support within the field. Increasing diversity in orthopaedics is essential for creating better training environments, improving patient care, and reducing health disparities, while also tackling the structural and cultural barriers that prevent a more inclusive and supportive culture for all surgeons [37, 45]. Mental health challenges highlight the need for resilience building, especially in high-risk specialties. Burnout and depression are key contributing factors, with orthopaedic surgeons having the highest suicide rate among surgical specialties [46]. The stigma surrounding mental health in surgery deters many from seeking the necessary support, instead relying on temporary measures, such as active coping, planning, restraint, or acceptance, that fail to address root causes [47].

Gender disparities in work–home conflicts, burnout, and career satisfaction identified differences between male and female surgeons. Female surgeons, often younger with fewer children, face more work–home conflicts, prioritising work over personal life despite similar hours to their male counterparts. This leads to higher burnout, depression, and lower work–life satisfaction. While both genders face similar burnout risks, female surgeons tend to sacrifice personal lives for their careers, increasing stress and psychological strain [11].

### Maladaptive Coping Strategies

Female surgeons encounter distinct challenges within their professional environments that drive maladaptive coping strategies. As described above, they experience higher overwork and real or perceived social stress compared to their male counterparts, with many expressing feelings of isolation and a lack of recognition in their roles [48]. These stressors are compounded by the pressures of balancing family responsibilities, rendering women particularly susceptible to chronic stress. Early-career female surgeons often resort to maladaptive coping mechanisms such as downplaying the severity of stress and engaging in distractions. While these strategies may provide temporary relief, they fail to address the underlying causes of stress and may ultimately exacerbate the problem over time [48].

Coping strategies are different depending on the surgeon’s experience: more experienced female surgeons tend to display self-pity, self-accusation, and avoidance, reflecting deeper struggles with stress that can contribute to burnout and emotional exhaustion [21, 48]. Alcohol abuse is a significant issue, closely linked to burnout, depression, and potential surgical and medical errors. Surgeons experiencing higher levels of burnout are more likely to develop alcohol use disorders, which in turn lowers career satisfaction and quality of life. Among U.S. surgeons, 15.4% met the criteria for alcohol use disorders, with female surgeons showing higher rates (25.6%) compared to their male counterparts (13.9%) [49]. Female surgeons are also more likely than their male counterparts to withdraw from interactions and engage in self-soothing behaviours, which are associated with lower coping effectiveness and heightened stress levels [39].

It is difficult to fully assess the extent of maladaptive coping strategies, as these behaviours are often accompanied by stigma and taboo which would lead an individual to hide or underplay them. Issues such as alcohol misuse, self-doubt, disordered eating, or other self-destructive coping mechanisms may be underreported due to concerns about professional repercussions and societal judgement [1, 50].

### Resilience and Coping

Resilience is defined as the ability to respond to stress in a healthy way, “bounce back,” and grow stronger from adversity [6]. Mindfulness has been proven to be an effective tool for reducing stress and improving mental health. Studies have shown that resilience can be built through mindfulness, self-monitoring, setting boundaries, and maintaining a positive attitude towards challenges [6, 51]. Mindfulness practices have been shown to significantly reduce stress, anxiety, and burnout among orthopaedic surgery trainees, with female surgeons benefiting the most. These findings highlight the importance of integrating mindfulness training into surgical education to provide better coping strategies, especially for women. Institutional support is crucial to turning individual efforts into lasting change. While stigma and denial may persist, early education and accessible support can help shift the culture. Mindfulness and peer networks empower women to thrive despite challenges, contributing to a resilient surgical workforce. A recent study demonstrated that even modest use of a mindfulness-based phone application led to significant reductions in stress, anxiety, and burnout among orthopaedic surgery residents, suggesting that simple, accessible tools can play a key role in supporting mental well-being [52].

While these stressors and barriers continue to persist, individual psychological resilience development offers a valuable pathway to mitigate the impact. Gender differences in coping strategies following surgical errors reveal that male and female surgeons approach these challenges in distinct ways [53]. Female surgeons were more likely to focus on calming themselves but reported less effective coping compared to their male counterparts. In addition, women were more likely to withdraw after an error, which may increase their risk of burnout. In contrast, male surgeons were more inclined to discuss errors with patients and colleagues, believing that these coping strategies enhanced performance [53]. Male surgeons have been found to seek help from friends or colleagues, while female surgeons prefer professional counsellors, which may affect the timeliness on which they gain support and deal with distress [54]. These differences may be shaped by subconscious gendered stereotypes that undermine the perceived coping ability. Compounding this, female surgeons continue to receive lower patient ratings in areas such as competence, medical knowledge, and technical skills, reflecting external biases rather than capability [55, 56]. Despite these feelings of inadequacy, in a meta-analysis that compared patients’ outcomes associated with surgeon’s gender, female surgeons had lower postoperative mortality rates compared to male surgeons, suggesting that their clinical skills are equal to or better than their male counterparts [57]. This challenges the perception that female surgeons lack skill, reinforcing that gender does not determine surgical competence.

Female surgeons often report exclusion and constant scrutiny, leading to feelings of isolation and self-doubt [50]. They advocate for improved conflict resolution strategies, including direct approaches, clearer reporting processes, and policies that address recurring issues. In addition, separating interpersonal conflicts from patient safety reporting is seen as essential for creating a fairer and more supportive work environment. This highlights the need for institutional reforms to better address microaggressions and workplace conflicts, ensuring that women and minority groups are more supported and retained in the field [58, 59].

There is a significant gap in the literature regarding targeted stress management interventions specifically for female surgeons [47]. Without early intervention and more robust education about the mental health challenges faced by surgeons, this crisis will continue to threaten the well-being of surgeons and undermine the future of a resilient surgical workforce.

## Conclusion

Women in surgery face distinct gender-specific stressors, including gender bias, harassment and disparities in training and academic opportunities, which significantly impact their mental health and professional satisfaction. These challenges are compounded by systemic barriers such as workplace culture and inadequate institutional support, perpetuating an environment that hinders their well-being and career progression. Moreover, the literature reveals a notable gap in understanding the specific coping mechanisms and resilience strategies female surgeons employ to navigate these obstacles, underscoring the need for dedicated investigation in this area.

Despite these persistent challenges, resilience offers a promising avenue for improvement. Practices such as mindfulness and peer support have demonstrated potential in reducing stress, anxiety, and burnout, particularly among female surgeons. However, the development and application of support structures tailored to their unique experiences remain underexplored. In particular, there is a lack of data from underrepresented countries and rural healthcare systems, which limits current generalisability; minimal longitudinal studies following surgeons across their careers; and few comprehensive studies evaluating targeted interventions addressing the root cause of mental health distress. Continued research and policy reforms are essential for fostering a more inclusive, supportive, and equitable surgical profession. By prioritising these efforts, the field can empower current and future generations of female surgeons to thrive, paving the way for a stronger and more diverse workforce. 

## Data Availability

Not applicable to this study.
