# Correction: Women in Surgery: An Analysis of Mental Health, Stress Perception and Resilience

**DOI:** 10.1007/s43465-025-01586-0

**Published:** 2025-10-08

**Authors:** Suzen Agharia, Hannah de Wet, Claudia Di Bella

**Affiliations:** 1 Faculty of Medicine, Nursing and Health Sciences, Monash University, Melbourne, VIC Australia; 2 School of Nursing and Midwifery, Faculty of Health, Deakin University, Melbourne, VIC Australia; 3 Orthopaedic Department, Sarcoma Unit, St Vincent’s Hospital Melbourne, Melbourne, VIC Australia; 4 Department of Surgery, The University of Melbourne, Melbourne, VIC Australia

**Correction: Indian Journal of Orthopaedics** 10.1007/s43465-025-01448-9

The original online version of this article was revised:

In this article, the authors’ names were incorrectly published as Agharia, M.S. and de Wet, M.H. The correct names should be Agharia, S. and de Wet, H.

The original article has been corrected.

